# Time-lapse image analysis reveals trigger-dependent differences in ASC speck lifetime in the NLRP3 inflammasome

**DOI:** 10.1038/s41598-026-50936-x

**Published:** 2026-05-04

**Authors:** M. Herring, A. Persson, R. Karlsson, D. Repsilber, M. Ejdebäck, E. Särndahl, N. Nikaein, O. Kotlyar

**Affiliations:** 1 School of Medical Sciences, Faculty of Medicine and Health, Örebro University, Örebro, Sweden; 2 Inflammatory Response and Infection Susceptibility Centre (iRiSC), Örebro University, Örebro, Sweden; 3 School of Bioscience, Systems Biology Research Centre, University of Skövde, Skövde, Sweden; 4 Department of Clinical Microbiology, Sahlgrenska University Hospital, Gothenburg, Sweden; 5 Department of Infectious Diseases, Institute of Biomedicine, Sahlgrenska Academy, University of Gothenburg, Gothenburg, Sweden; 6 Nanoxis Consulting AB, Gothenburg, Sweden; 7 Man-Technology-Environment Research Center (MTM), School of Science and Technology, Örebro University, Örebro, Sweden; 8 Robot Navigation & Perception Lab (RNP), School of Science and Technology, Örebro University, Örebro, Sweden; 9 AI, Robotics and Cybersecurity Center (ARC), Örebro University, Örebro, Sweden

**Keywords:** Biomarkers, Computational biology and bioinformatics, Immunology

## Abstract

Formation of the NLRP3 inflammasome occurs in response to a wide range of triggers and leads to pyroptosis and release of IL-1β and IL-18. This has widely been considered an “all or nothing” response leading to similar cellular outcomes each time, once the inflammasome has formed, a view that has been challenged over the years. This assumption has largely been based on endpoint measurements at the population level, producing metrics that fail to adequately capture dynamic responses. Herein, we utilize live-cell imaging combined with an algorithmic approach to track individual ASC-GFP specks over time, formed in response to the triggers ATP, monosodium urate and nigericin. Using this approach, we report trigger-dependent differences in speck lifetime. The use of the proposed algorithm requires a relatively small dataset, while still providing insights into NLRP3 inflammasome dynamics downstream of inflammasome activation at the single-cell level.

**Supplementary Information:**

The online version contains supplementary material available at 10.1038/s41598-026-50936-x.

## Introduction

The inflammatory response is a central feature of innate immunity, and inflammasomes play an important role in this response. Inflammasomes are multimeric protein complexes that form in response to exogenous or endogenous substances to facilitate the activation and release of the proinflammatory cytokines interleukin (IL)-1β and IL-18^1^. First described in 2002^2^, inflammasomes have been revealed to play a crucial role in immune homeostasis. The nod-like receptor family pyrin domain containing 3 (NLRP3) inflammasome, which has become the most extensively studied inflammasome, forms in response to a large number of different Pathogen-Associated Molecular Pattern (PAMP) and Damage-Associated Molecular Pattern (DAMP) triggers, including adenosine triphosphate (ATP)^3^, certain crystals including monosodium urate (MSU)^4–7^, bacterial exotoxins^8^, various metals^9^, and viral dsRNA^10^.

NLRP3 inflammasome triggers act by either induction of ion flux, mediation of lysosomal damage, inhibition of the electron transport chain^11^ and/or oxidative phosphorylation. ATP and nigericin both lead to changes in ion flux, albeit through different mechanisms. ATP binds the P2X7 receptor, an ion-gated channel that leads to potassium ion (K^+^) efflux and sodium and calcium ion influx^12^. Nigericin is an ionophore that is membrane-impermeant until bound to either a hydrogen ion (H^+^) or potassium ion (K^+^). Thus, nigericin binds extracellular H^+^ and transports it into the cytosol. It can then bind cytosolic K^+^, which is transported out of the cell, leading to K^+^ efflux, H^+^ influx, and concurrent cytosolic acidification^13^. MSU is a crystal that, when taken up by the cell, leads to lysosomal damage^14^, release of lysosomal components, and subsequent NLRP3 inflammasome activation^15^.

One of the hallmarks observed during the activation of multiple types of inflammasomes is the formation of one or more large protein complexes, or specks, consisting of a sensor (e.g. NLRP3) and apoptosis-associated speck-like protein containing a CARD (ASC)^16^. The ASC-speck functions as a scaffold for recruitment of the other components into the multi-protein inflammasome complex, subsequently resulting in activation and release of cytokines (e.g. IL-1β and IL-18) and proinflammatory gasdermin D-mediated cell death, or pyroptosis^17,18^.

The most commonly used NLRP3 inflammasome readouts, including ASC-speck formation, extracellular IL-1β/IL-18 concentration, and LDH release, display population level inconsistencies in their temporal association when inflammasome formation is induced via different triggers^19^. Experimental end-point analysis that quantifies these readouts (e.g. cytokine/LDH concentration or number of ASC-specks) often compares populations, which renders the assessment of associations between inflammasome readouts difficult, as individual cells’ contribution to each readout cannot be determined. Research has however been moving more towards single-cell analysis utilizing imaging and flow cytometry techniques to assess connections between cellular events during, among other things, inflammasome activation^20–22^. The use of single-cell techniques allows for tracking of readouts from individual cells, and provides a clearer link between cellular-level readouts, as exemplified in, for instance, single-cell experiments investigating IL-1β secretion dynamics in human monocytes^22^.

After ASC fusion to green fluorescent protein (GFP), the formed ASC-GFP-speck can be readily observed by fluorescence microscopy as a large (~ 1 μm), relatively circular, high intensity particle^23^, making tracking of each speck possible. This opens the possibility to study speck dynamics at the single-cell level from a time series of images where individual specks are identified and tracked over time. Cell lines containing the ASC-GFP fusion are commercially available, and the human monocyte THP1 cell line is frequently used in inflammasome research^24–26^ and readily forms ASC-GFP-specks upon receiving a priming and activation signal. THP1-ASC-GFP cells contain a plasmid with ASC-GFP under the control of an NF-κB promoter. Thus, when cells are primed with a priming signal, like LPS, NF-κB is activated which results in expression of ASC-GFP in addition to endogenously expressed ASC. ASC-GFP is subsequently incorporated into ASC-specks upon inflammasome complex formation, facilitating speck detection and quantitation.

Particle detection and tracking is commonly performed by one or a combination of two approaches: either “classical” algorithms or deep learning (DL) techniques^27^, each with their advantages and disadvantages. Crucially, algorithm-based technologies require careful optimization of detection parameters to ensure correct detection, whereas DL approaches require massive, labeled training sets^28^. The latter, however, is often not obtainable in experimental biological settings. Moreover, DL-based approaches for object tracking rely on learned patterns or embeddings^29,30^. This makes it complicated or even impossible to employ DL approaches for tracking the specks or particles since they lack distinguishable visual patterns.

To investigate inconsistencies previously observed at the population level when assessing inflammasome activation, the aim of this paper is to study the trigger-dependency of ASC-GFP speck lifetime in THP-1-ASC-GFP cells and to assess if the duration of the speck itself shows variability in regard to different triggers. This was done by tracking individual ASC-GFP-specks in a single-cell analysis approach of inflammasome readouts, using previously obtained fluorescence images^19^. An algorithm-based approach was utilized to investigate differences in lifetime depending on the trigger activating the ASC-GFP-speck formation.

## Methods

Cell culture, treatments and imaging were performed previously^19^. Briefly, 3 × 10^4^ THP-1-ASC-GFP cells per well in 96-well imaging plates (Agilent Technologies, Santa Clara, CA) were differentiated with 0.1 µg/mL phorbol myristate acetate (PMA) for 24 h, followed by a resting period of 24 h. The human THP1-ASC-GFP monocyte cell line was purchased from Invivogen (Invivogen, San Diego, CA). Five experimental replicates of each treatment were plated in duplicate wells. Cells were primed with 500 ng/mL ultrapure LPS (Invivogen, San Diego, CA) for 4 hours, and triggering of inflammasome complex formation was performed by addition of either 5 mM ATP (Merck, Darmstadt, Germany), 100 µg/mL MSU (Invivogen, San Diego, CA) or 10 µM nigericin (Invivogen, San Diego, CA). The triggers ATP, MSU, and nigericin were chosen as they are all commonly used for NLRP3 inflammasome activation^19,31–33^. Live cell imaging was performed using the EVOS™ M7000 Imaging System (Invitrogen, Carlsbad, CA) with On Stage Incubator equipped with an EVOS 10x fluorite objective and a GFP LED Cube, with an autofocusing step performed in the transmission channel. Twelve adjacent, but non-overlapping, fields of view were imaged in the center of each well every 30 min for 24 h. This generated time series of 16-bit greyscale TIFF images (maximum pixel intensity 4,095) consisting of 49 timepoints (including starting zero point) and a total of 588 images per well, resulting in 17,640 frames.

### Speck detection and tracking

**Fig. 1:**
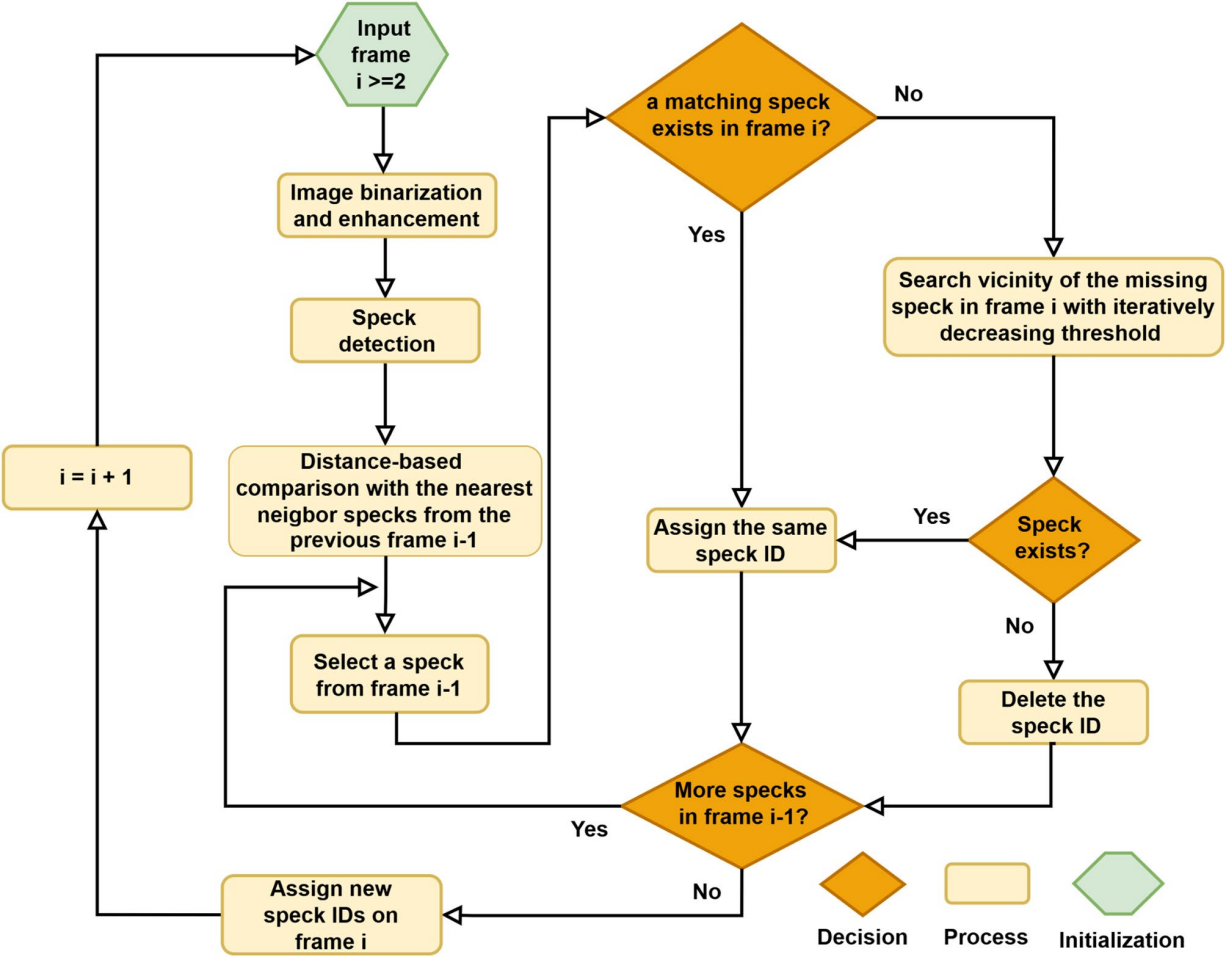
ASC-GFP-speck detection and tracking algorithm. Flowchart illustrates steps of detecting and updating specks from frame to frame.

A flowchart summarizing the proposed speck detection and tracking algorithm is presented in Fig. 1. In the first step, a global detection algorithm was applied to the collected images. Images were binarized using a first intensity threshold (int_thr1_ - see section parameter estimation) and were then enhanced by a dilation followed by an erosion filter (kernel size = 5). At the dilation step, a pixel was set to be part of the background only if all pixels under the kernel were part of the background. This filter thereby eliminates noise within the speck area and connects disjointed speck areas if they exist. However, the dilation step also increases the speck area which is compensated by the subsequent application of an erosion filter. At the erosion step, a pixel is considered to be part of the background if at least one pixel under the kernel is part of the background. The processed images were then explored for small continuous clusters of pixels (1 < cluster area < 120; equivalent to a circle with a radius of ~ 9.9 μm) of high circularity (≥ 80%), which were considered as ASC-GFP-specks.

For continued detection and tracking of ASC-GFP specks, the following procedure was implemented: in each iteration of the algorithm, specks were detected in the current frame and were then compared to the identified specks in the previous frame. The nearest neighbor ASC-GFP-specks between the two frames were considered to be the same speck if their distance was less than a predefined distance threshold (d_max_ - see section parameter estimation). This threshold marks the maximum distance an ASC-GFP-speck can move between two consecutive frames i.e., within 30 min.

If no speck was detected in the current frame within d_max_ distance of a speck in the previous frame, a local search algorithm was implemented. The local search algorithm takes the coordinates of a speck and investigates the vicinity of these coordinates in the next frame for any speck-like objects using a second, lower intensity threshold (int_thr2_ - see section parameter estimation). This is to account for the reduction of GFP signal after speck formation and the problems with autofocusing (see limitations). In the square region (patch) centered around the speck’s center, with side length equal to half of the maximum movement distance d_max_, binarization is performed using a second, lower threshold int_thr2_. If no speck is detected within the patch, the secondary threshold is reduced by 25, otherwise if more than one speck is detected, the second threshold is increased by 25. These iterations are repeated until exactly one speck is detected in the patch, or until a maximum of ten iterations is reached. If, after local search, no specks or more than one speck were detected, the original speck was classified as disappeared.

Newly formed ACS-GFP specks are only identified using the first intensity threshold int_thr1_, as these new specks have high intensity. Specks which were detected in a frame but could not be attributed to any specks in the previous frame were indexed as newly formed specks. For speck detection and tracking self-developed Python scripts were implemented. The code was initially inspired by Kukil^34^ and Canu, S^35^.

### Parameter estimation

Values of three main algorithm parameters - the first intensity threshold (int_thr1_), the second intensity threshold (int_thr2_), and the maximum distance (number of pixels) an ASC-GFP-speck can move between frames within a 30-minute interval (d_max_) - were estimated using manually collected data. The parameter d_max_ was estimated by manually measuring the movement of 415 specks between consecutive frames. The manual measurement was performed in FIJI^36^ by manually annotating specks in consecutive frames. A script written in ImageJ’s built-in macro language (Supplementary 1) was subsequently used to measure the distance moved by individual specks. The maximum movement was calculated and rounded up to 40 pixels, based on the collected data (Supplementary 2). Notably, 97.83% of all measurements fell below d_max_/2. So, the local search algorithm restricts its search to an area around the speck’s center, with side length equal to d_max_/2.

**Fig. 2:**
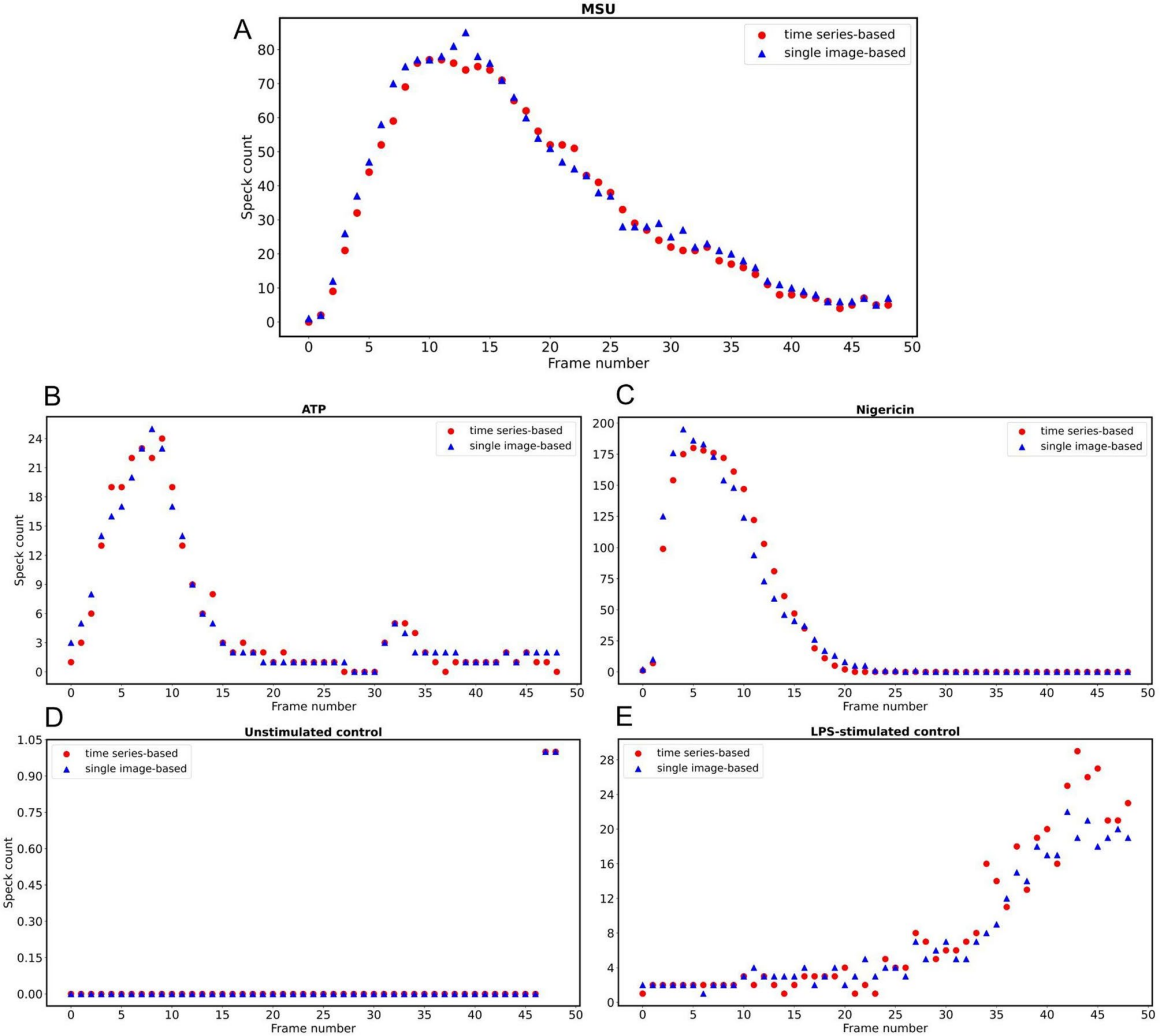
Parameter estimation. **(A)** ASC-GFP-specks in PMA-differentiated, LPS-primed THP-1 cells were counted both using FIJI (blue triangles) and the proposed algorithm (red circles) for a randomly selected field of view in one of the MSU-triggered wells. Algorithm parameters were selected to minimize the difference between the counts obtained from the two methods. **(B-E)** The estimated values were assessed by comparing the counts obtained from FIJI and the proposed algorithm for one randomly selected well triggered by either ATP or nigericin as well as for untreated controls and LPS-primed controls.

The first intensity threshold *int*_*thr1*_ is the intensity threshold used in the global speck detection algorithm. Objects with intensity more than *int*_*thr1*_ are detected as ASC-GFP-specks if they fulfill predefined size and circularity conditions. The second intensity threshold *int*_*thr2*_ is the intensity threshold used in the local speck detection algorithm. By defining *int*_*thr2*_ < *int*_*thr1*_, we tend to keep tracking those ASC-GFP-specks in which the GFP signal gradually decreases below *int*_*thr1*_ over time. To estimate *int*_*thr1*_ and *int*_*thr2*_, ASC-GFP-specks from PMA-differentiated, LPS-primed THP-1 cells were counted using FIJI in a randomly selected dataset comprising 49 consecutive frames, after triggering with MSU as previously described^19^. The thresholds *int*_*thr1*_ = 2100 and *int*_*thr2*_ = 1300 were selected to minimize the difference between the counts performed by FIJI and the proposed algorithm (Fig. 2.A). To further validate the estimated parameters, the proposed algorithm was used to count ASC-GFP-specks in two randomly selected time series, one each from ATP or nigericin-treated cells. Each time series consisted of 49 consecutive frames. Results were then compared to counts performed using FIJI (Fig. 2.B-C). The Normalized Root Mean Square Error (NRMSE) was calculated to be 4.3%, 5.3%, and 4.9% for MSU, nigericin and ATP, respectively. The controls (Fig. 2. D-E) have also been included for comparison, even though these conditions were not used in the further analysis of speck lifetime.

### Data analysis

For each inflammasome trigger - ATP, MSU and nigericin – lifetimes of ASC-GFP-specks were calculated using the proposed algorithm across five plates, each consisting of two wells per trigger. Each well contained 12 fields of view, and each field comprised 49 frames. ASC-GFP-specks lifetimes from 12 fields of view were aggregated for each well, resulting in ten datasets (five plates, two wells per plate) for each trigger. Subsequently, the two datasets from each plate were merged, yielding five final datasets per trigger. These datasets were then used to statistically analyze the lifetime distribution and speck count for each trigger.

Differences in lifetime distribution were compared using two approaches. First, speck lifetimes for the three triggers were compared based on 90% quantiles of lifetime distributions observed during the duration of the experiment, as this allows for a more stable assessment of long lifetimes than reporting the (single) maximum lifetime. The significance of differences was tested using the Wilcoxon Rank Sum test (two-sided, 5% significance level). Second, Kaplan-Meier survival analysis was implemented and differences between survival curves for the three triggers were compared based on the log-rank statistics; both overall hypothesis of identical survival curves and pairwise comparisons were implemented. Differences in total speck number between triggers were analyzed by one-way ANOVA with Tukey multiple comparisons test. p-values below 0.05 were considered statistically significant. Data and self-developed Python analysis scripts will be made available upon publication.

## Results and discussion

### The proposed algorithm successfully detects and tracks ASC-GFP-specks independent of the trigger

**Fig. 3:**
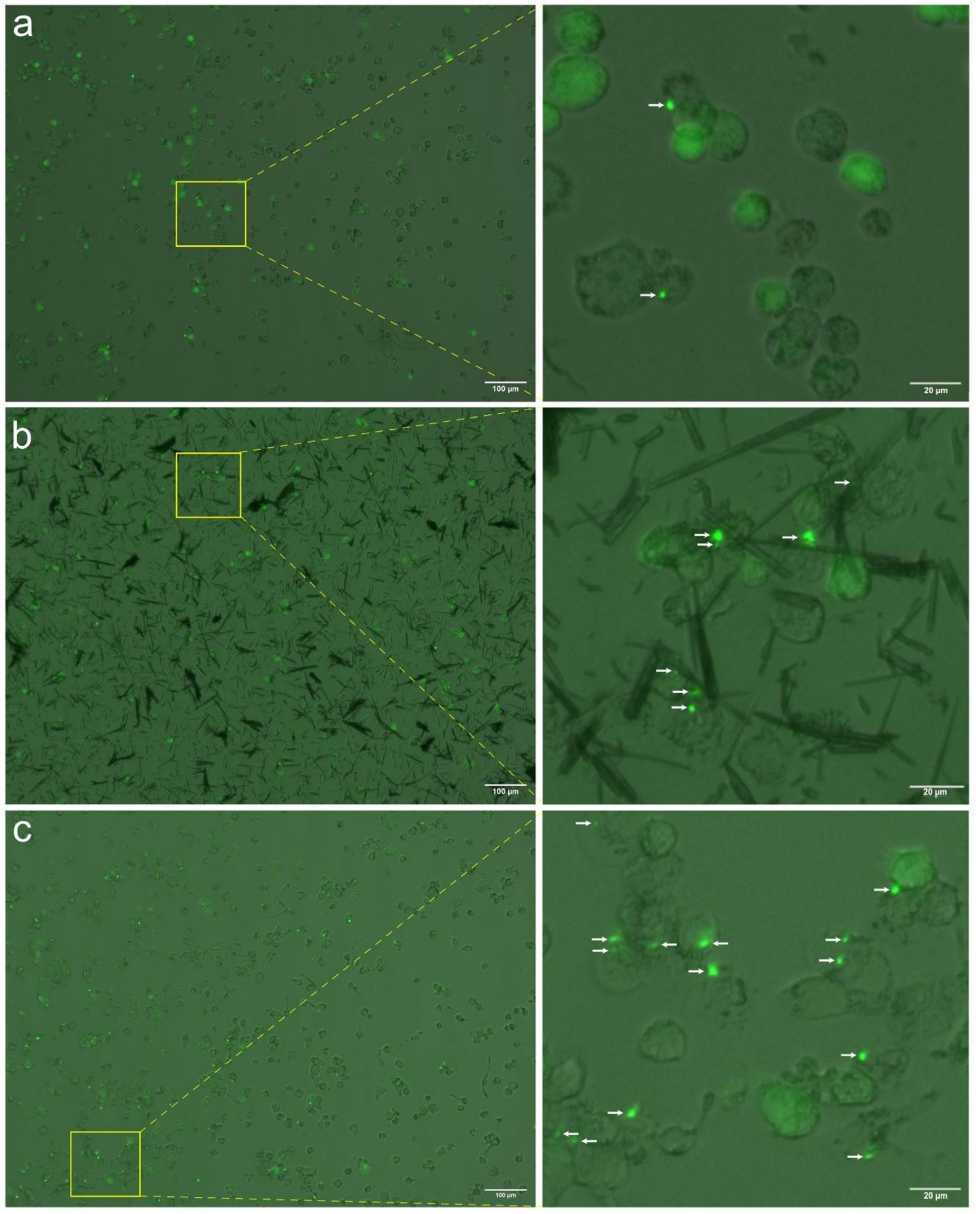
Speck formation in THP-1 cells. Speck formation in PMA-differentiated, LPS-primed THP-1-ASC-GFP cells after triggering with either **(A)** ATP, **(B)** MSU or **(C)** nigericin. Specks (white arrows) are seen as bright, green spots. Figure shows representative images taken 1 h after addition of trigger; inserts are chosen to show ASC-GFP-speck formation. Transmission and GFP channels have been merged, with the GFP channel pseudo-colored green.

The analysis of various NLRP3 inflammasome readouts is a basis for inflammasome-related research. One commonly utilized readout is the formation of an ASC-speck, a large, cytosolic structure formed as a consequence of NLRP3 inflammasome priming and activation (Fig. 3). Although ASC-speck formation is a typical occurrence during NLRP3-inflammasome activation, it does not automatically entail functional inflammasome activation^37^. Nonetheless, speck formation is still an important readout within the field of inflammasome research. Quantification of ASC-specks, often through imaging techniques, grants population level metrics about speck formation and can be utilized to assess the ability of compounds to promote or inhibit inflammasome assembly. Here, the detection and tracking algorithm proposed in the Methods section was utilized to analyze how the human monocyte THP1 cell line responds to different triggers regarding inflammasome speck formation.

**Fig. 4:**
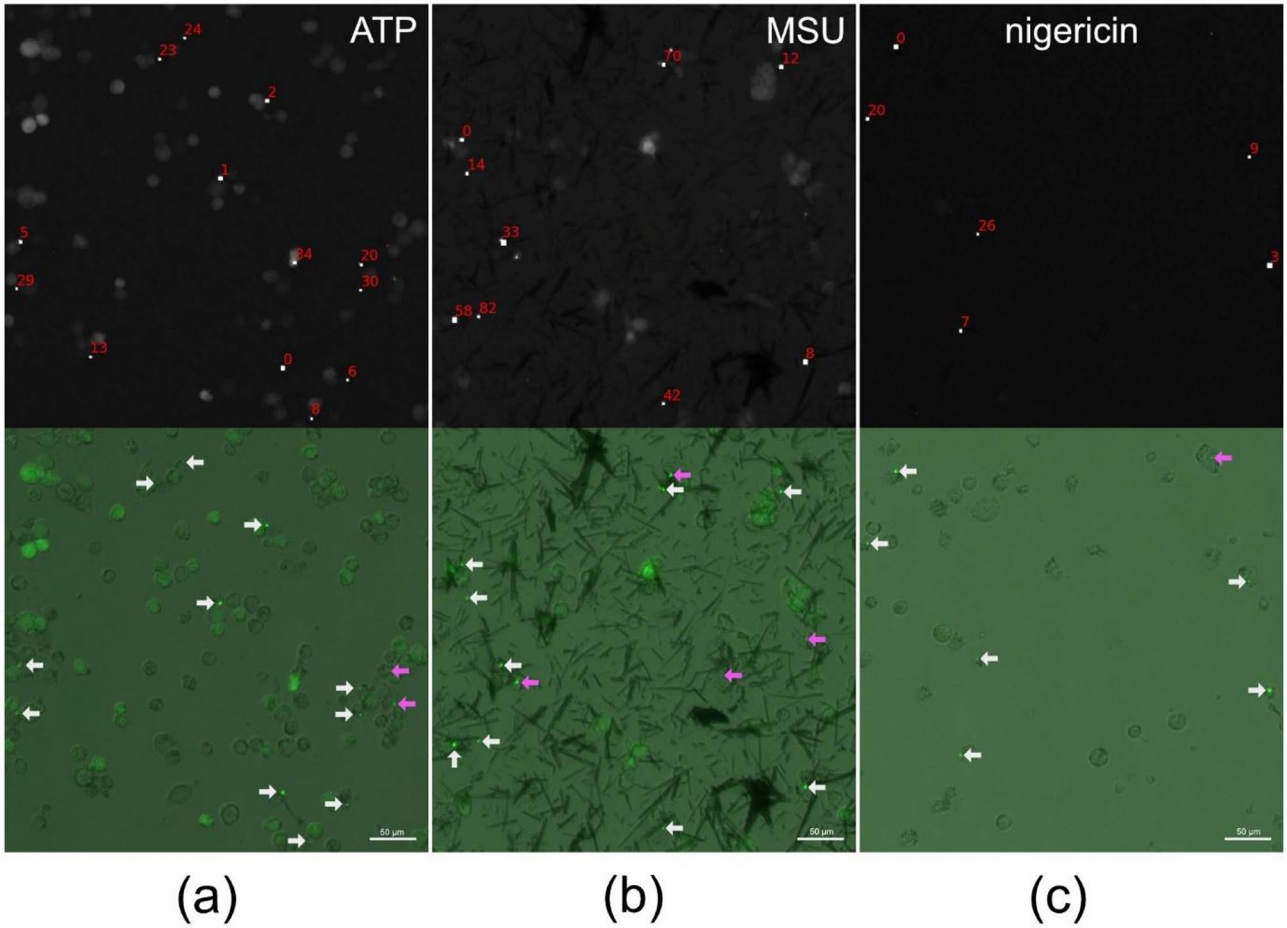
Speck detection in PMA-differentiated LPS-primed THP-1-ASC-GFP cells 2 h after the addition of **(A)** ATP, **(B)** MSU and **(C)** nigericin. ASC-GFP-specks are automatically identified and indexed and a bounding box is added. White arrows point to visually identifiable specks, which are detected successfully. Pink arrows point to the visually identifiable specks, which were not identified by the algorithm since they failed to meet the predefined intensity, size and/or circularity criteria. The figure shows representative images. Scale bars are 50 μm.

The algorithm was able to detect the vast majority of specks, failing only to detect those whose intensity, size or circularity lies outside the predefined ranges (Fig. 4). It did not however consistently detect fewer specks than the single-image based approach (Fig. 2), nor did we find incorrectly annotated specks, indicating that while our algorithmic approach may fail to identify specks that do not meet the selected parameter criteria, it is able to detect specks that are missed by the single-image approach. Overall, the number of specks detected with the two approaches displays a high level of similarity (Fig. 2).

### Speck lifetime and time of formation differ with trigger

We next applied the algorithm to track individual ASC-GFP-specks over time and assessed the number of specks formed during the course of the experiment, as well as the lifetime of individual specks formed in response to different inflammasome triggers. Identified ASC-GFP-specks with a lifetime of one frame (< 60 min) were deemed as artifacts and were removed from subsequent analyses, as the majority of specks observed with just a one-frame lifetime were found to be false positives upon manual inspection. Visual inspection revealed several causes for the misidentification. Out-of-focus specks changing size or shape from frame to frame, specks getting obscured by crystals in consecutive frames, specks appearing in the final frame, and intensity of faint specks dropping below the detection threshold right after detection, mislead the algorithm to register a speck as a very short lived one.

**Fig. 5:**
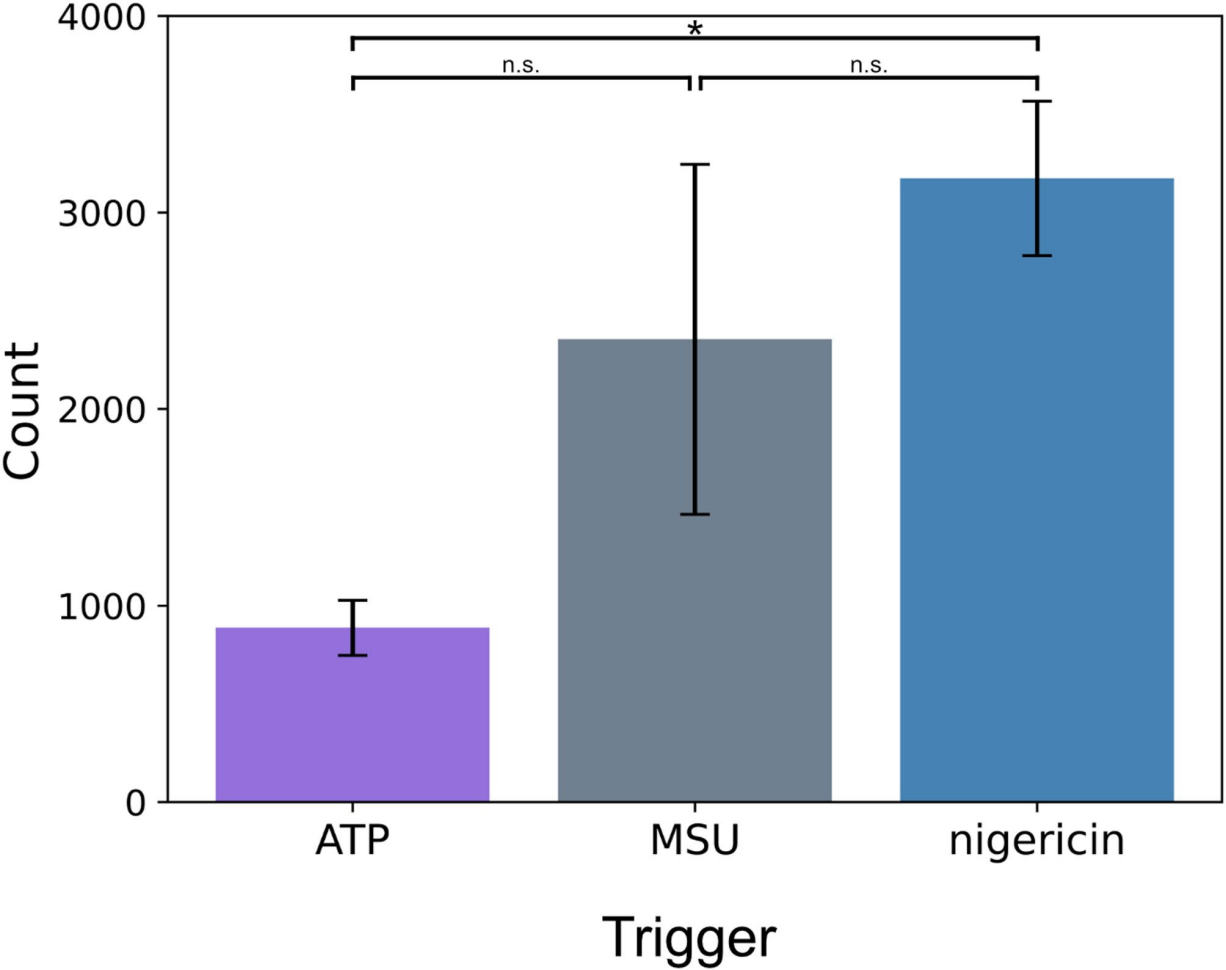
Individual ASC-GFP-speck count. Mean number across the experiments of individual ASC-GFP-specks formed over 24 h in PMA-differentiated, LPS-primed THP-1-ASC-GFP cells after triggering speck formation with either ATP, MSU or nigericin. The mean was calculated using the sum of individual specks numbers from all fields of view for each biological replicate (see section Data analysis). ASC-GFP-specks were tracked over 24 h and quantified. Data were analyzed by one-way ANOVA with Tukey multiple comparisons test and are shown as mean ± SEM, *n* = 5. *p-adj < 0.05, n.s.: not significant.

Individual specks were quantified to determine the number of individual ASC-GFP-specks formed over 24 h, regardless of timepoint. While we have previously quantified mean ASC-GFP-speck numbers at individual timepoints at the population level^19^, individual speck-tracking allows quantification of the number of specks formed throughout the experiment, without counting the same specks multiple times. This approach shows that triggering speck formation with nigericin induced significantly more specks than ATP (p. adj = 0.036), with MSU stimulation causing an intermediate number of specks during the initial 24 h after triggering (Fig. 5). The number of individual specks formed is in line with our previous, population-based, results^19^.

**Fig. 6:**
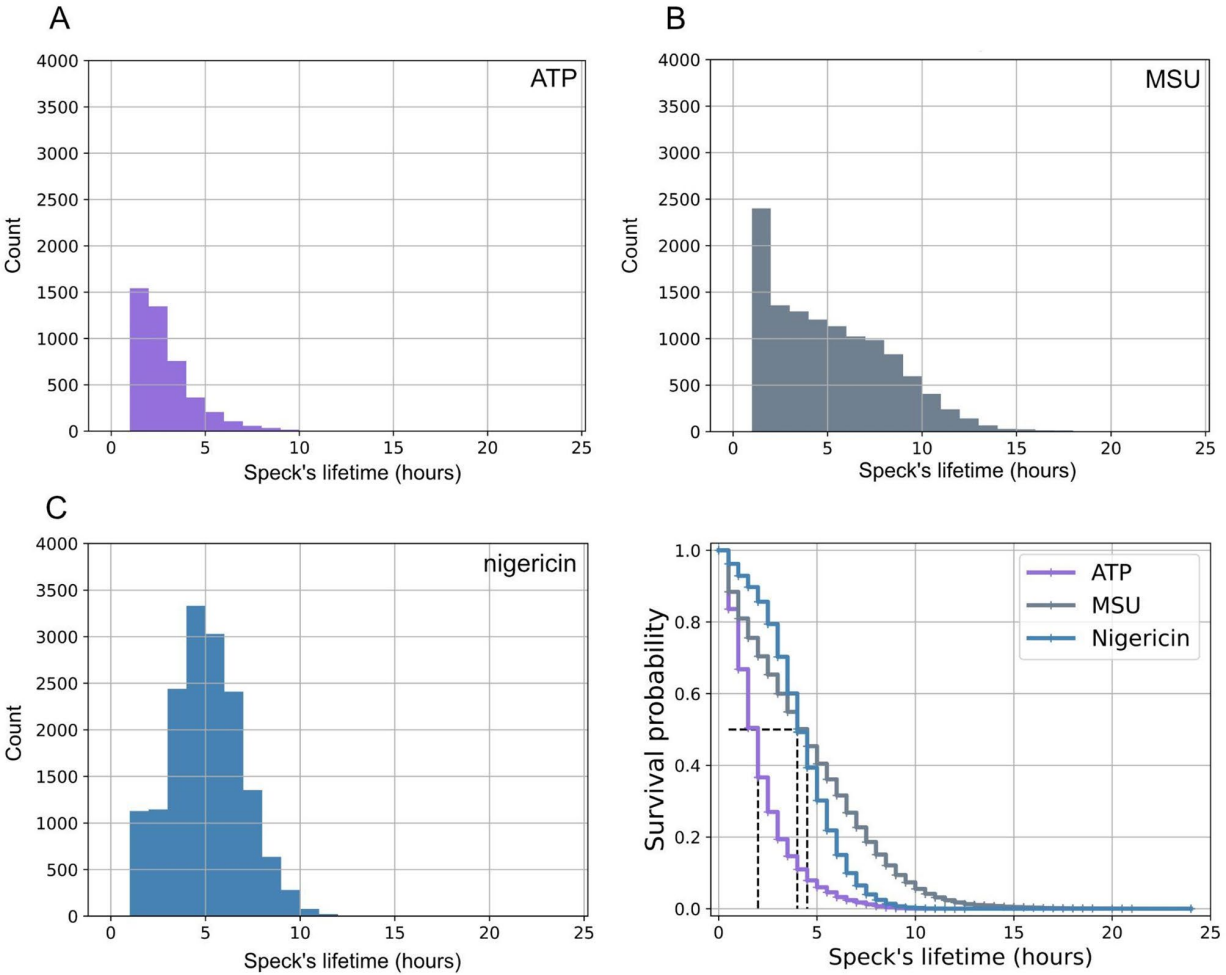
ASC-GFP-speck lifetime. Specks from PMA-differentiated, LPS-primed THP-1 cells were automatically identified and tracked after triggering with either (**A**) ATP, (**B**) MSU or (**C**) nigericin. (**D**) Survival curves of specks appearing throughout the experiment for different triggers. For each trigger, data from all fields of view in all replicates (*n* = 5) were pooled and analyzed. The results were then truncated to eliminate the specks with a life cycle of 1 frame as artifacts. Dashed lines in (D) show median survival speck’s lifetime that is equal to 2.0, 4.5, and 4.0 h for ATP, MSU, and nigericin respectively.

Histograms of the lifetime of ASC-GFP-specks illustrate different underlying distributions of speck lifetime for different triggers (Fig. 6). The distribution of speck lifetimes after triggering with either ATP or MSU were skewed to the left, whereas speck lifetime after triggering with nigericin had a bell-shaped distribution. Further, the 90% quantile of speck lifetimes (after removal of specks with a lifetime of one frame) were compared (Wilcoxon ranksum test, *n* = 5) and were significantly different between MSU and ATP (p-value = 0.012) as well as nigericin and ATP (p-value = 0.009) indicating that both MSU and nigericin tend to result in longer maximum speck lifetimes than ATP. No significant difference in speck lifetime was detected between MSU and nigericin (p-value = 0.754).

**Fig. 7:**
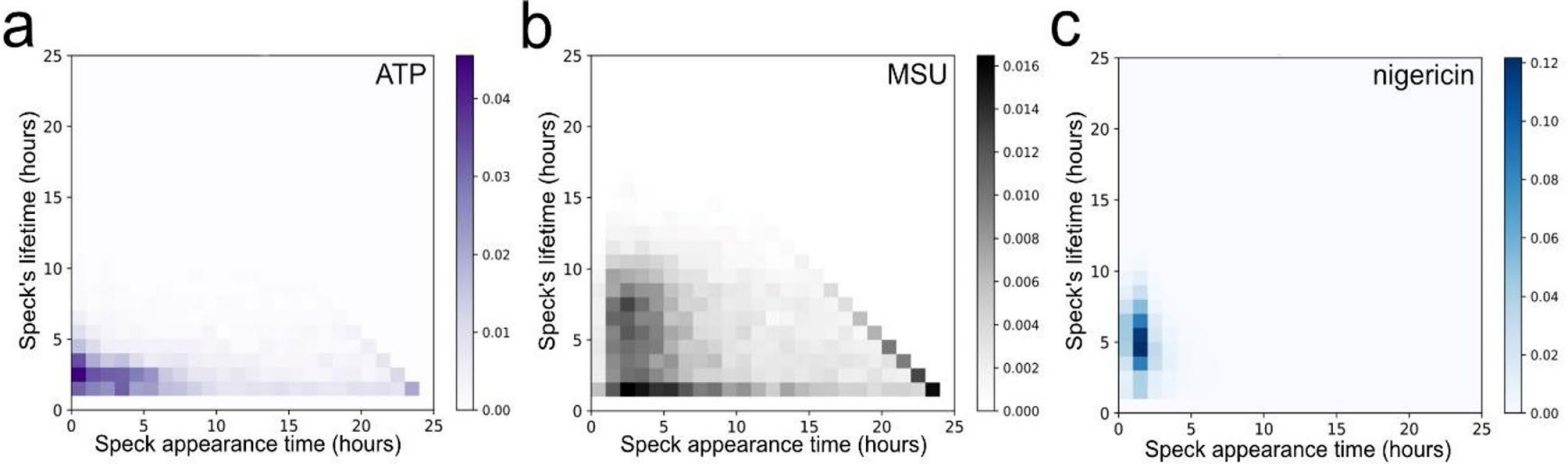
Time of formation and lifetime of ASC-GFP-specks. Relative number of specks formed at each timepoint and their duration, normalized to the total number of specks for each trigger (*n* = 5). THP-1-ASC-GFP cells were PMA-differentiated, primed with LPS and triggered with either (**A**) ATP, (**B**) MSU or (**C**) nigericin. The diagonal seen for ATP- and MSU-triggered cells corresponds to the sum of all specks remaining at the end of the experiment.

We next investigated the time of formation of specks and their lifetime to see if any connection between the two could be observed (Fig. 7). Cells triggered with ATP (Fig. 7A) seemed to predominantly form ASC-GFP-specks during the first 5 h post-activation, while the speck formation in MSU-triggered cells (Fig. 7B) was slightly more spread out in time. Nigericin-triggered cells almost exclusively formed ASC-GFP-specks within the first three hours (Fig. 7C). The results do not indicate that there is any connection between a speck´s lifetime and its time of formation. However, the significant Kaplan-Meier log-rank test (ATP-MSU, p-value = 2 × 10^− 16^; ATP-nigericin, p-value < 2 × 10^− 16^; MSU-nigericin, p-value < 2 × 10^− 16^) for the null hypothesis of identical survival curves for all triggers supports that the lifetime of ASC-GFP-specks (irrespective of time of formation) differs between triggers (Fig. 6D).

Nigericin, a K^+^ ionophore, induces robust and sustained potassium efflux and cytosolic acidification, which might lead to rapid assembly of stable, longer-lived ASC-specks even though the rapid and robust cell death induced by nigericin may lead to premature quenching of GFP, thus artificially shortening speck lifetime. While premature quenching of GFP is a possibility with all of the triggers utilized, the effect may be greater with triggers that induce rapid cell death, thus distorting the difference in observed speck lifetime between triggers. ATP activates P2X7 receptors, resulting in transient K^+^ efflux lasting a few minutes before the receptor desensitizes or ATP is degraded in the extracellular space, which could mean shorter-lived specks^3,38,39^. MSU crystals, through phagocytosis and lysosomal damage, may initiate a slower but prolonged activation, giving rise to longer-lived specks that form continuously over time. Recent studies have shown that NLRP3 undergoes phase separation, forming liquid-like or gel-like condensates whose dynamics vary depending on the stimulus. Time-dependent solidification of ASC/NLRP3 specks may contribute to their increased longevity following nigericin or MSU stimulation, compared to ATP-induced specks that remain more dynamic and are rapidly resolved^40,41^. Post-translational modifications of ASC, cytosolic pH changes, and interactions with other effector proteins may also influence speck longevity^42^. Triggers of NLRP3 inflammasome formation such as ATP, nigericin, alum and silica also induce post-translational modifications of NLRP3 including palmitoylation, phosphorylation and ubiquitination that can both facilitate inflammasome formation, or promote degradation of NLRP3 by the proteasome, through autophagy-mediated degradation or chaperone-mediated autophagy^43–45^. Furthermore, triggers of NLRP3 inflammasome formation have been shown in Cell Painting experiments to induce additional, trigger-specific effects in cells^46^, but it is unclear what these effects are or if they impact inflammasome formation, function or degradation in any way. Further, it is important to note that ASC-speck formation is not immediately equatable with inflammasome formation^37^, and that speck formation and inflammasome effector function are, or can be, decoupled in multiple inflammasome types^47,48^. Thus, the importance of variations in ASC-speck lifetime with regards to inflammasome functionality remains to be determined.

**Fig. 8:**
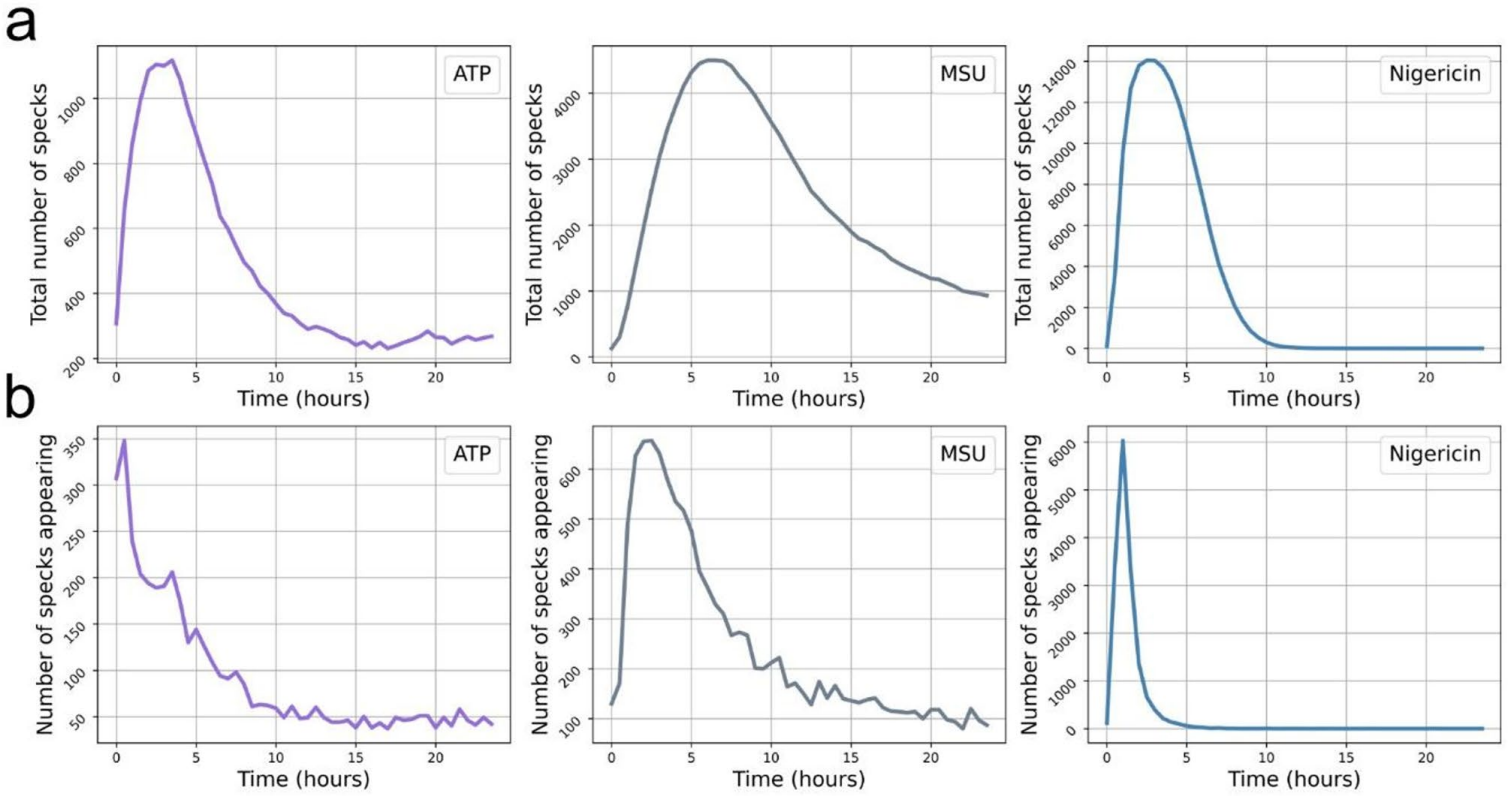
Total number of specks present and number of specks formed at each timepoint. ASC-GFP-specks in PMA-differentiated, LPS-primed THP-1-ASC-GFP cells were automatically detected and tracked. The total number of ASC-GFP-specks present at each timepoint (**A**) and number of ASC-GFP-specks formed at each timepoint (**B**) after triggering speck formation with either ATP, MSU or nigericin. Data is the sum of all fields of view from all experimental replicates (*n* = 5).

By quantifying and tracking individual ASC-GFP-specks, we can confirm previous reports that speck formation begins shortly after addition of trigger (Figs. 7A-C and 8B)^19,49^.

The algorithm-based detection method used in this study differs from the detection methods we have utilized previously (FastRandomForest classifier with the training features Hessian matrix, Sobel filter difference of gaussians and membrane projections, followed by intensity thresholding and filtering by size). Despite this, the curves for speck numbers are highly similar^19^, supporting that a relatively simple algorithm can adequately detect ASC-GFP-specks. The ability to track specks also reveals that although speck formation is highest in the first 5 h after activation with all three triggers, triggering speck formation with ATP or MSU leads to continuous formation of specks, which is not the case after triggering with nigericin (Fig. 8A). Further, ATP-triggered speck formation results in shorter-lived specks than triggering with either MSU or nigericin. This illustrates distinct kinetics of ASC-GFP-specks, namely rapidly forming longer lived specks (nigericin), continuously forming longer lived specks (MSU) and continuously forming shorter lived specks (ATP) (Fig. 7). The highest number of specks present is 3–4 h after triggering with ATP or nigericin and 6–8 h after triggering with MSU (Fig. 8B). Likely the differences described are due to the induction route of each trigger as it is well described that the amplitude of inflammasome readouts, on a population basis, differs among triggers^38^ dependent on the main mediator being ion-fluxes, acidification, involvement of electron transport chain or lysosomal component. This description of longevity and time-related aspects of inflammasome function may represent a first insight into the different involvements of how inflammasome activation contributes to both health and disease states.

### Experimental limitations

The ability to track objects in a series of images is dependent on the possibility to clearly distinguish the object of interest. Imaging of 3-dimensional, biological systems comes with a multitude of difficulties, including keeping the object(s) in focus and handling obstruction by other structures in the system being imaged. Out-of-focus imaging of fluorescent punctae (e.g. ASC-GFP-specks) often results in flaring (Supplementary Fig. 1), making the speck appear larger and less defined, and also lowers the intensity signal. While the latter is not a problem initially, the lowered signal level may lead to premature loss of signal, potentially shortening the time during which the speck is detectable. Further, the introduced variation in speck size, shape and intensity can hamper parameter estimation. Obstruction of specks may cause loss of tracking due to an inability to detect the obstructed speck, or it may introduce artifacts (Supplementary Fig. 2) that may affect speck detection and tracking. Furthermore, termination of the experiment may lead to shortened speck lifetime, especially with triggers that cause late speck formation and/or longer-lived specks.

### Algorithmic and data analysis limitations

The combination of ASC-GFP-speck movement and the appearance of new specks (occurring between frames) creates potential issues during speck tracking. While this has been addressed with the assumption that the closest speck to the position of a speck in a previous image is the same speck, it is possible that a new speck is formed closer to the original position of a speck. This can lead to mislabeling of both specks. Taking images with higher temporal resolution may alleviate this by minimizing the movement distance but may lead to increased risk of photobleaching and phototoxicity. For more robust speck tracking, additional information regarding individual speck’s shape, size, and pixel intensities is required. This may facilitate building of embeddings that could be utilized to match the specks through the time series of images. In such a case, DL approaches for speck tracking could alleviate problems arising from speck obscurement or in cases where a speck appears within the movement distance of another speck. Additionally, mapping cells to specks can enhance the robustness of speck tracking with DL-based methods. The cell contains more distinguishable information and patterns in comparison to specks, making it easier to track than an ASC-GFP-speck. Using DL in this study is however hampered by data-limitations. For our future studies, we plan to address this problem by creating larger datasets, also complementing them with brightfield images.

## Conclusions

By measuring the formation and lifetime of individual specks, we reveal trigger-dependent differences in ASC-GFP-speck lifetime not measurable by end-point analysis. The observed differences may reflect trigger-specific mechanisms of speck formation which impact the outcomes of inflammasome formation and/or degradation and facilitate inflammatory responses tailored to distinct insults. We show that the tracking of ASC-GFP-specks is possible with a relatively simple algorithmic approach, requiring substantially less data than deep learning approaches. Further, this approach is suitable for smaller-scale experiments and allows for the generation of time series data at the single-cell level. This is another step in the direction towards evaluation of inflammasome readouts at the single-cell level, which is required for future, more precise, elucidation of inter-readout associations downstream of inflammasome activation.

## Electronic Supplementary Material

Below is the link to the electronic supplementary material.

Supplementary Material 1

Supplementary Material 2

Supplementary Material 3

## Data Availability

The data that support the findings of this study are available from the corresponding author upon reasonable request. Original code is publicly accessible at [https://github.com/niloofar-nikaein/Speck\_detection\_and\_tracking](https:/github.com/niloofar-nikaein/Speck_detection_and_tracking) .
