# Supplementary material for: Time-lapse image analysis reveals trigger-dependent differences in ASC speck lifetime in the NLRP3 inflammasome
Source: Sci Rep. 2026 May 4;16:14173. doi: 10.1038/s41598-026-50936-x (PMC13139490; doi:10.1038/s41598-026-50936-x)
Supplement: Supplementary file 1 — Supplementary Material 1 [file 41598_2026_50936_MOESM1_ESM.docx]

# Supplementary data 1

## Distance measurment script

Written in .ijm macro language for ImageJ (FIJI). Note that a stack of paired images containing marked specks (e.g. using the Multipoint tool) is required. A speck is first annotated in image one of the stack, then image two, before a new speck is annotated.

Macro “Name of Macro” {

getSelectionCoordinates(x, y);

getPixelSize(unit, pw, ph);

n = nResults;

for (i = 0; i < x.length; i += 2) {

if (i + 1 < x.length) {

dx = (x[i+1] - x[i]) * pw;

dy = (y[i+1] - y[i]) * ph;

distance = sqrt(dx*dx + dy*dy);

setResult("Distance", n++, distance);

print("Distance between point " + (i + 1) + " and point " + (i + 2) + ": " + distance + " " + unit);

}

}

updateResults();

}

# Supplementary data 2

Distance measured

| **1st image timepoint** | **2nd image timepoint** | **distance (pixels)** |
| --- | --- | --- |
| p01 | p02 | 23.854 |
| p02 | p03 | 7.071 |
| p02 | p03 | 4.485 |
| p02 | p03 | 7.341 |
| p02 | p03 | 2.603 |
| p02 | p03 | 4.069 |
| p02 | p03 | 15.413 |
| p02 | p03 | 6.146 |
| p02 | p03 | 4.269 |
| p02 | p03 | 7.180 |
| p02 | p03 | 15.585 |
| p02 | p03 | 5.270 |
| p02 | p03 | 25.979 |
| p02 | p03 | 10.883 |
| p02 | p03 | 5.270 |
| p02 | p03 | 2.000 |
| p02 | p03 | 3.543 |
| p02 | p03 | 8.007 |
| p02 | p03 | 4.643 |
| p02 | p03 | 4.922 |
| p02 | p03 | 11.552 |
| p02 | p03 | 1.000 |
| p02 | p03 | 6.263 |
| p02 | p03 | 9.944 |
| p02 | p03 | 4.738 |
| p02 | p03 | 10.995 |
| p02 | p03 | 12.494 |
| p02 | p03 | 7.454 |
| p02 | p03 | 7.311 |
| p02 | p03 | 6.128 |
| p02 | p03 | 4.216 |
| p02 | p03 | 6.412 |
| p02 | p03 | 5.667 |
| p02 | p03 | 6.289 |
| p02 | p03 | 5.467 |
| p02 | p03 | 5.821 |
| p02 | p03 | 8.280 |
| p02 | p03 | 5.831 |
| p02 | p03 | 3.145 |
| p02 | p03 | 5.696 |
| p02 | p03 | 6.667 |
| p02 | p03 | 2.687 |
| p02 | p03 | 35.025 |
| p02 | p03 | 5.735 |
| p02 | p03 | 5.088 |
| p02 | p03 | 6.119 |
| p02 | p03 | 13.569 |
| p02 | p03 | 8.360 |
| p02 | p03 | 6.872 |
| p02 | p03 | 8.544 |
| p02 | p03 | 12.293 |
| p02 | p03 | 6.864 |
| p02 | p03 | 5.821 |
| p02 | p03 | 6.839 |
| p02 | p03 | 3.887 |
| p02 | p03 | 6.037 |
| p02 | p03 | 7.063 |
| p02 | p03 | 5.207 |
| p03 | p04 | 1.581 |
| p03 | p04 | 1.803 |
| p03 | p04 | 2.500 |
| p03 | p04 | 2.372 |
| p03 | p04 | 5.590 |
| p03 | p04 | 2.828 |
| p03 | p04 | 3.010 |
| p03 | p04 | 3.162 |
| p03 | p04 | 5.927 |
| p03 | p04 | 2.658 |
| p03 | p04 | 3.553 |
| p03 | p04 | 4.031 |
| p03 | p04 | 5.303 |
| p03 | p04 | 2.462 |
| p03 | p04 | 2.926 |
| p03 | p04 | 1.677 |
| p03 | p04 | 4.123 |
| p03 | p04 | 4.243 |
| p03 | p04 | 9.100 |
| p03 | p04 | 2.550 |
| p03 | p04 | 0.559 |
| p03 | p04 | 15.301 |
| p03 | p04 | 2.610 |
| p03 | p04 | 11.071 |
| p03 | p04 | 13.250 |
| p03 | p04 | 0.791 |
| p03 | p04 | 4.138 |
| p03 | p04 | 3.000 |
| p03 | p04 | 2.704 |
| p03 | p04 | 2.761 |
| p03 | p04 | 13.509 |
| p03 | p04 | 1.250 |
| p03 | p04 | 2.358 |
| p03 | p04 | 2.610 |
| p03 | p04 | 2.795 |
| p03 | p04 | 2.372 |
| p03 | p04 | 15.598 |
| p03 | p04 | 36.125 |
| p03 | p04 | 2.236 |
| p03 | p04 | 3.579 |
| p03 | p04 | 3.363 |
| p03 | p04 | 2.610 |
| p03 | p04 | 5.772 |
| p03 | p04 | 8.559 |
| p03 | p04 | 2.704 |
| p03 | p04 | 1.521 |
| p03 | p04 | 2.693 |
| p03 | p04 | 2.761 |
| p03 | p04 | 1.677 |
| p03 | p04 | 3.400 |
| p03 | p04 | 2.236 |
| p03 | p04 | 15.782 |
| p03 | p04 | 1.953 |
| p03 | p04 | 2.704 |
| p13 | p14 | 0.707 |
| p13 | p14 | 0.707 |
| p13 | p14 | 0.000 |
| p13 | p14 | 0.000 |
| p13 | p14 | 0.000 |
| p13 | p14 | 0.373 |
| p13 | p14 | 0.601 |
| p13 | p14 | 0.333 |
| p13 | p14 | 0.833 |
| p13 | p14 | 0.373 |
| p13 | p14 | 0.236 |
| p13 | p14 | 2.500 |
| p13 | p14 | 0.236 |
| p13 | p14 | 0.898 |
| p13 | p14 | 0.333 |
| p13 | p14 | 0.250 |
| p13 | p14 | 0.000 |
| p13 | p14 | 0.750 |
| p13 | p14 | 0.471 |
| p13 | p14 | 0.745 |
| p13 | p14 | 0.333 |
| p13 | p14 | 0.471 |
| p13 | p14 | 0.943 |
| p13 | p14 | 1.054 |
| p13 | p14 | 0.472 |
| p13 | p14 | 0.745 |
| p13 | p14 | 0.471 |
| p13 | p14 | 0.000 |
| p13 | p14 | 0.333 |
| p13 | p14 | 0.333 |
| p13 | p14 | 0.333 |
| p13 | p14 | 0.333 |
| p13 | p14 | 0.667 |
| p13 | p14 | 0.667 |
| p09 | p10 | 1.374 |
| p09 | p10 | 0.333 |
| p09 | p10 | 0.471 |
| p09 | p10 | 0.471 |
| p09 | p10 | 0.667 |
| p09 | p10 | 0.333 |
| p09 | p10 | 0.471 |
| p09 | p10 | 0.707 |
| p09 | p10 | 0.500 |
| p09 | p10 | 2.500 |
| p09 | p10 | 1.000 |
| p09 | p10 | 1.000 |
| p09 | p10 | 0.687 |
| p09 | p10 | 1.118 |
| p09 | p10 | 0.500 |
| p09 | p10 | 8.500 |
| p09 | p10 | 0.667 |
| p09 | p10 | 1.374 |
| p09 | p10 | 0.943 |
| p09 | p10 | 0.745 |
| p09 | p10 | 1.202 |
| p09 | p10 | 0.333 |
| p09 | p10 | 1.374 |
| p09 | p10 | 0.745 |
| p09 | p10 | 0.667 |
| p09 | p10 | 0.667 |
| p09 | p10 | 0.333 |
| p09 | p10 | 1.202 |
| p09 | p10 | 1.491 |
| p09 | p10 | 0.745 |
| p09 | p10 | 1.118 |
| p09 | p10 | 9.962 |
| p09 | p10 | 2.500 |
| p09 | p10 | 1.000 |
| p09 | p10 | 1.000 |
| p09 | p10 | 0.559 |
| p09 | p10 | 0.707 |
| p09 | p10 | 1.118 |
| p09 | p10 | 1.118 |
| p09 | p10 | 1.000 |
| p09 | p10 | 0.500 |
| p09 | p10 | 0.500 |
| p09 | p10 | 0.500 |
| p09 | p10 | 1.000 |
| p09 | p10 | 1.414 |
| p09 | p10 | 0.500 |
| p09 | p10 | 0.500 |
| p09 | p10 | 1.118 |
| p09 | p10 | 0.000 |
| p04 | p05 | 2.236 |
| p04 | p05 | 2.062 |
| p04 | p05 | 2.500 |
| p04 | p05 | 1.803 |
| p04 | p05 | 2.500 |
| p04 | p05 | 1.581 |
| p04 | p05 | 1.581 |
| p04 | p05 | 5.025 |
| p04 | p05 | 1.803 |
| p04 | p05 | 1.803 |
| p04 | p05 | 1.118 |
| p04 | p05 | 1.581 |
| p04 | p05 | 1.491 |
| p04 | p05 | 1.886 |
| p04 | p05 | 2.749 |
| p04 | p05 | 1.886 |
| p04 | p05 | 1.491 |
| p04 | p05 | 1.333 |
| p04 | p05 | 2.828 |
| p04 | p05 | 1.491 |
| p04 | p05 | 1.491 |
| p04 | p05 | 3.333 |
| p04 | p05 | 1.491 |
| p04 | p05 | 2.404 |
| p04 | p05 | 4.472 |
| p04 | p05 | 12.445 |
| p04 | p05 | 4.773 |
| p04 | p05 | 1.886 |
| p04 | p05 | 1.667 |
| p04 | p05 | 2.134 |
| p04 | p05 | 0.667 |
| p04 | p05 | 1.414 |
| p04 | p05 | 2.134 |
| p04 | p05 | 0.745 |
| p04 | p05 | 1.333 |
| p04 | p05 | 2.404 |
| p04 | p05 | 2.028 |
| p04 | p05 | 1.886 |
| p04 | p05 | 1.944 |
| p04 | p05 | 2.236 |
| p04 | p05 | 3.771 |
| p04 | p05 | 1.667 |
| p04 | p05 | 1.054 |
| p04 | p05 | 2.357 |
| p04 | p05 | 2.028 |
| p04 | p05 | 11.511 |
| p04 | p05 | 1.581 |
| p04 | p05 | 3.202 |
| p04 | p05 | 8.201 |
| p04 | p05 | 3.808 |
| p04 | p05 | 1.118 |
| p04 | p05 | 2.915 |
| p04 | p05 | 2.236 |
| p04 | p05 | 2.693 |
| p04 | p05 | 1.581 |
| p04 | p05 | 1.414 |
| p06 | p07 | 3.162 |
| p06 | p07 | 3.606 |
| p06 | p07 | 0.000 |
| p06 | p07 | 6.403 |
| p06 | p07 | 5.523 |
| p06 | p07 | 1.000 |
| p06 | p07 | 2.062 |
| p06 | p07 | 1.414 |
| p06 | p07 | 3.041 |
| p06 | p07 | 1.118 |
| p06 | p07 | 7.500 |
| p06 | p07 | 1.581 |
| p06 | p07 | 13.124 |
| p06 | p07 | 28.151 |
| p06 | p07 | 25.298 |
| p06 | p07 | 6.325 |
| p06 | p07 | 2.062 |
| p06 | p07 | 1.500 |
| p06 | p07 | 2.062 |
| p06 | p07 | 39.275 |
| p06 | p07 | 2.693 |
| p06 | p07 | 2.000 |
| p06 | p07 | 1.000 |
| p06 | p07 | 3.606 |
| p06 | p07 | 4.924 |
| p06 | p07 | 8.944 |
| p06 | p07 | 1.581 |
| p06 | p07 | 2.000 |
| p06 | p07 | 24.683 |
| p06 | p07 | 1.803 |
| p06 | p07 | 2.550 |
| p12 | p13 | 0.850 |
| p12 | p13 | 0.667 |
| p12 | p13 | 14.907 |
| p12 | p13 | 1.491 |
| p12 | p13 | 2.981 |
| p12 | p13 | 2.000 |
| p12 | p13 | 1.886 |
| p12 | p13 | 5.696 |
| p12 | p13 | 18.523 |
| p12 | p13 | 1.333 |
| p12 | p13 | 0.000 |
| p12 | p13 | 2.749 |
| p12 | p13 | 2.108 |
| p12 | p13 | 0.000 |
| p12 | p13 | 0.667 |
| p12 | p13 | 14.981 |
| p12 | p13 | 1.333 |
| p12 | p13 | 2.981 |
| p12 | p13 | 0.000 |
| p12 | p13 | 12.401 |
| p12 | p13 | 2.108 |
| p12 | p13 | 0.000 |
| p12 | p13 | 9.357 |
| p12 | p13 | 0.943 |
| p12 | p13 | 6.667 |
| p12 | p13 | 1.491 |
| p12 | p13 | 1.491 |
| p12 | p13 | 3.333 |
| p12 | p13 | 0.667 |
| p12 | p13 | 2.404 |
| p12 | p13 | 2.981 |
| p12 | p13 | 3.333 |
| p12 | p13 | 0.667 |
| p12 | p13 | 0.000 |
| p12 | p13 | 1.333 |
| p12 | p13 | 0.667 |
| p12 | p13 | 0.000 |
| p12 | p13 | 0.943 |
| p12 | p13 | 0.943 |
| p12 | p13 | 14.757 |
| p12 | p13 | 12.293 |
| p12 | p13 | 0.667 |
| p12 | p13 | 0.943 |
| p12 | p13 | 0.667 |
| p12 | p13 | 0.000 |
| p12 | p13 | 0.667 |
| p12 | p13 | 0.667 |
| p12 | p13 | 3.399 |
| p12 | p13 | 0.667 |
| p12 | p13 | 6.864 |
| p12 | p13 | 1.886 |
| p12 | p13 | 0.943 |
| p12 | p13 | 0.667 |
| p12 | p13 | 0.943 |
| p12 | p13 | 6.289 |
| p12 | p13 | 3.590 |
| p12 | p13 | 1.886 |
| p12 | p13 | 0.667 |
| p12 | p13 | 0.000 |
| p01 | p02 | 4.007 |
| p01 | p02 | 3.887 |
| p01 | p02 | 1.795 |
| p01 | p02 | 28.241 |
| p01 | p02 | 4.776 |
| p01 | p02 | 4.177 |
| p01 | p02 | 12.649 |
| p02 | p03 | 10.512 |
| p02 | p03 | 13.744 |
| p02 | p03 | 5.498 |
| p02 | p03 | 3.590 |
| p02 | p03 | 2.981 |
| p02 | p03 | 11.333 |
| p02 | p03 | 4.269 |
| p02 | p03 | 4.055 |
| p02 | p03 | 4.216 |
| p02 | p03 | 12.293 |
| p02 | p03 | 5.375 |
| p02 | p03 | 3.333 |
| p02 | p03 | 11.870 |
| p02 | p03 | 5.207 |
| p02 | p03 | 5.963 |
| p02 | p03 | 7.180 |
| p02 | p03 | 4.216 |
| p02 | p03 | 4.269 |
| p02 | p03 | 7.775 |
| p02 | p03 | 2.749 |
| p02 | p03 | 6.146 |
| p02 | p03 | 8.537 |
| p02 | p03 | 8.485 |
| p08 | p09 | 2.953 |
| p08 | p09 | 0.000 |
| p08 | p09 | 0.667 |
| p08 | p09 | 8.110 |
| p08 | p09 | 0.667 |
| p08 | p09 | 0.667 |
| p08 | p09 | 2.749 |
| p08 | p09 | 1.491 |
| p08 | p09 | 0.667 |
| p08 | p09 | 0.667 |
| p08 | p09 | 0.943 |
| p08 | p09 | 1.491 |
| p08 | p09 | 6.000 |
| p08 | p09 | 3.771 |
| p08 | p09 | 8.110 |
| p08 | p09 | 0.943 |
| p08 | p09 | 1.333 |
| p08 | p09 | 0.943 |
| p08 | p09 | 0.667 |
| p08 | p09 | 11.175 |
| p08 | p09 | 1.333 |
| p08 | p09 | 0.667 |
| p08 | p09 | 0.667 |
| p08 | p09 | 0.943 |
| p14 | p15 | 0.559 |
| p14 | p15 | 0.333 |
| p14 | p15 | 0.943 |
| p14 | p15 | 0.667 |
| p14 | p15 | 1.333 |
| p14 | p15 | 2.358 |
| p14 | p15 | 0.667 |
| p14 | p15 | 4.069 |
| p14 | p15 | 0.471 |
| p14 | p15 | 17.586 |
| p14 | p15 | 1.067 |
| p14 | p15 | 1.414 |
| p14 | p15 | 0.167 |
| p08 | p09 | 0.250 |
| p08 | p09 | 0.500 |
| p08 | p09 | 15.670 |
| p08 | p09 | 0.745 |
| p08 | p09 | 1.500 |
| p08 | p09 | 1.346 |
| p08 | p09 | 4.853 |
