# Supplementary material for: Time-lapse image analysis reveals trigger-dependent differences in ASC speck lifetime in the NLRP3 inflammasome
Source: Sci Rep. 2026 May 4;16:14173. doi: 10.1038/s41598-026-50936-x (PMC13139490; doi:10.1038/s41598-026-50936-x)
Supplement: Supplementary file 2 — Supplementary Material 2 [file 41598_2026_50936_MOESM2_ESM.docx]

# Supplementary figure 1


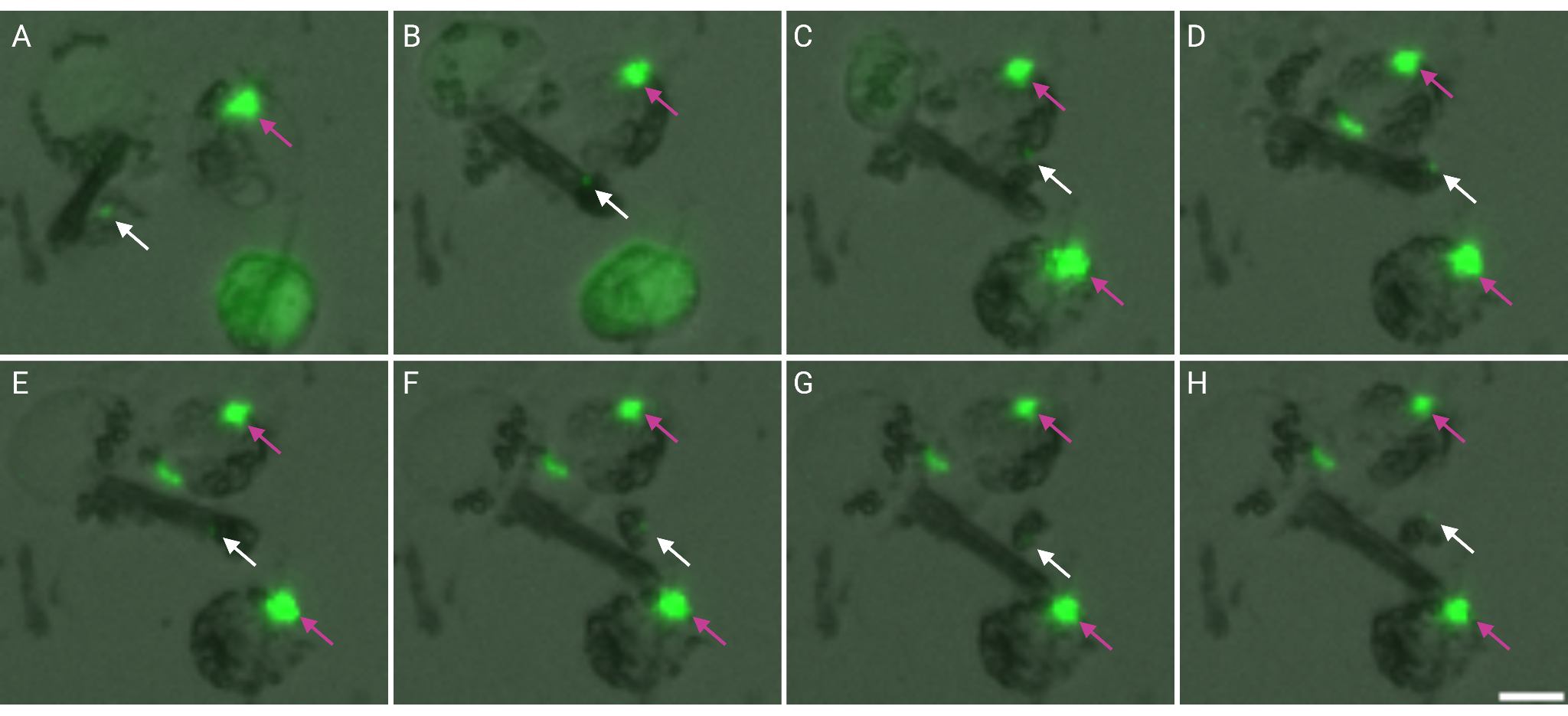


Supplementary figure 1. **Limitations during imaging.** Low-intensity ASC-GFP-speck (white arrows) followed through eight frames (3.5 hours). Speck becomes obscured by MSU crystal (B, D and E) and by cellular debris (F and G). Out-of-focus specks (pink arrows) can also be observed as large irregular punctae. Figure shows a representative series of images chosen to highlight limitations. Scale bar is 10 µm.
