# Supplementary material for: Time-lapse image analysis reveals trigger-dependent differences in ASC speck lifetime in the NLRP3 inflammasome
Source: Sci Rep. 2026 May 4;16:14173. doi: 10.1038/s41598-026-50936-x (PMC13139490; doi:10.1038/s41598-026-50936-x)
Supplement: Supplementary file 3 — Supplementary Material 3 [file 41598_2026_50936_MOESM3_ESM.docx]

# Supplementary figure 2


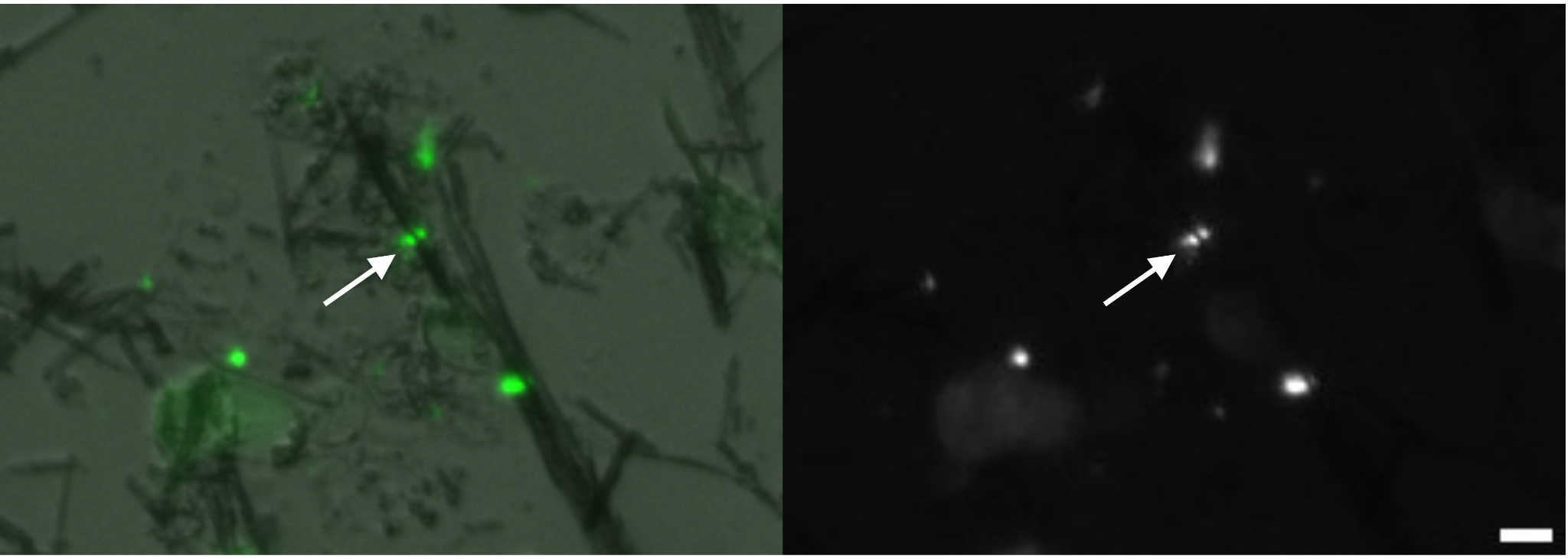


Supplementary figure 2: **Artifact created by obscurement of high-intensity ASC-GFP-speck.** What appears to be two ASC-GFP-specks (white arrow) is a single speck, partially obscured by an MSU crystal. Figure shows a representative image chosen to highlight limitations. Scale bar is 10 µm.
